# Redefinition and Measurement Dimensions of Sustainable Employability Based on the swAge-Model

**DOI:** 10.3390/ijerph182413230

**Published:** 2021-12-15

**Authors:** Jianwei Deng, Jiahao Liu, Wenhao Deng, Tianan Yang, Zhezhe Duan

**Affiliations:** 1 Beijing Institute of Technology, School of Management and Economics, Haidian District, Beijing 100081, China; Dengjianwei2006@163.com (J.D.); bitliujiahao@163.com (J.L.); dwenhao215@163.com (W.D.); 2 Sustainable Development Research Institute for Economy and Society of Beijing, Beijing 100081, China; 3 Institute of Urban Governance, School of Government, Shenzhen University, Shenzhen 215123, China

**Keywords:** sustainable employability, definition, measurement dimensions, the swAge-model

## Abstract

Objectives: To solve the labour shortage, we clarify the definition and dimensions of sustainable employability, and make it possible to develop sustainable employability scales in the future and lay the foundation for subsequent quantitative research. Finally, people’s sustainable employability can be improved. Highly sustainable employability employees can continue to work in the labour market and their working lives can be prolonged. Labour market supply will increase and labour shortage will be partly solved. Methods: We discuss the concept of sustainable employability based on some previous studies. Our conclusion is that the existing definitions and measurement dimensions are problematic. The swAge-model, a tool that helps us understand how to make working life more sustainable and healthier for all ages, can be the basis of sustainable employability. Results: We develop a discussion paper concerning the definition and measurement dimensions of sustainable employability using the swAge-model with an added factor of intrinsic work value and the dynamic chain. Conclusions: Our definition of sustainable employability takes environmental factors into consideration and makes it clear that it is not a solely personal characteristic, but the result of an interaction between individuals and the environment, thus distinguishing employability from work ability. We use the swAge-model as a basis to make the composition of our definition more logical and informed. Our measurement dimensions are clearly described to facilitate the future development of a scale, and our concept may ultimately help to extend the working lives of older and retired workers and thus solve the future labour shortage problem.

## 1. Introduction

Sustainable employability commonly refers to the ability of employees to participate in work and the labour market during their lifetimes [1]. Because this ability is very important for individuals, organisations, and society, it deserves our attention. For individuals, work provides economic security and social ties, as well as forming an important part of and giving meaning to their daily lives. Organisations need productive employees to improve organisational performance and survive market competition. Society needs as many people as possible to participate in the labour market to maintain economic welfare and social stability [2]. In view of the aging of the global population, there may be a labour shortage in the future [3] and research on sustainable employability is necessary. However, the current definition and framework of sustainable employability are still very confusing, which hinders subsequent research [4]. This article aims to solve this possible labour market crisis through clarifying definition and dimensions of employees’ sustainable employability and laying foundation for scales development and quantitative research that can affect this ability. Finally, sustainable employability can be achieved and prolong employees’ working lives, and even extend them past the official retirement age.

In the medical sector, for example, the aging of the labour force has led to a continuous decrease in the overall number of medical workers. At the same time, the number of patients has been increasing. This imposes a heavy workload on the medical staff, causing physical, mental, and emotional pressures [5]. More and more healthcare workers encounter mental health problems due to job difficulties, and many choose to change departments or leave their jobs before the official retirement age, thus causing a further reduction in medical staff [6]. Meanwhile, the productivity of medical workers may decline due to their poor physical condition and the instability of the staff caused by resignations and changes. The quality of medical services may decline accordingly [7]. The reduction of medical resources and the increase in demand are very urgent problems for the organisation of medicine as a whole and society. Sustainable employability can prolong the careers of employees in the medical industry, guarantee the working ability of the medical staff, retain trained experts, and maintain their irreplaceable skills and professional knowledge. Helping medical organisations build a stable and healthy workforce will benefit individuals, organisations, and society [8,9].

## 2. The Development and Deficiencies of the Sustainable Employability Concept

Although sustainable employability is of great significance to individuals, organisations, and society, there are still many gaps and deficiencies in the definition and dimensions used to measure this capacity. It was first defined by van der Klink et al. as follows: ‘sustainable employability means that, throughout their working lives, workers can achieve tangible opportunities in the form of a set of capabilities. They also enjoy the necessary conditions that allow them to make a valuable contribution through their work, now and in the future, while safeguarding their health and welfare. This requires, on the one hand, a work context that facilitates this for them and on the other, the attitude and motivation to exploit these opportunities [1]. This definition has been widely used in subsequent studies [9,10,11]. It describes sustainable employability as a multidimensional concept, acknowledges the importance of employees and job characteristics, and, to a certain extent, acknowledges the longitudinal characteristic, an individual’s employability over time, of this concept. However, Fleuren et al. noted that there are omissions in this definition. They argued that: 1. this definition does not specify which dimensions constitute an individual’s sustainable employability; 2. sustainable employability cannot be regarded as a characteristic of both the job and the employee simultaneously; 3. based on assumptions that have not been fully verified, we cannot assert that achieving value in work will lead to sustainable employability; 4. the definition of sustainable employability should include unemployed people to make the concept applicable to a larger group; and 5. the definition of sustainable employability should address the inherently longitudinal characteristic, that is, an individual’s employability over time [2]. Among the shortcomings proposed, the first is particularly obvious in later studies. For example, Roczniewska et al. and Hazelzet et al. both acknowledged the definition put forward by van der Klink when studying the theoretical background of sustainable employability, but in the quantitative analysis stage their measurement dimensions varied. The former mainly used three dimensions: productivity, physical and mental health, and happiness, while the latter replaced happiness with valuable work and long-term perspective [9,11]. In other words, because there are no clear measurement dimensions in the definition, different studies have different understandings of the same definition. Their measurement methods are biased and cannot be standardised, which makes it impossible to directly compare different research results and form a comprehensive understanding of the concept.

In their later research, Fleuren et al. formulated their own definition according to the points they raised, after integrating and reviewing existing sustainable employability definitions. They argued that sustainable employability refers to the ability of an individual to function in work and the labour market, or that employability is not negatively (and preferably positively) affected by personal employment status. This ability can be captured by combining nine indicators (perceived health status, work ability, recovery needs, fatigue, job satisfaction, job motivation, perceived employability, skill gap, and job performance) to describe the extent to which a person can be employed at different stages of his or her work life [4]. Compared with the deficiencies in the definition proposed by van der Klink et al., this definition clearly sets out nine dimensions of sustainable employability, which facilitates its measurement. The definition also emphasises that sustainable employability is a longitudinal concept with a time dimension. However, it remains unclear whether employability is a personal characteristic or the result of an interaction between the environment and the individual, and whether its definition covers reasonable groups.

Some studies argue that sustainable employability is not a personal concept, but the result of an interaction between the employee and the environment [12,13], while Fleuren et al. consider it a personal characteristic. We are more inclined towards the interactionist point of view, because sustainable employability should take into account labour market characteristics [14]. The concept of employment itself also includes personal and organisational characteristics as input variables, which is the important difference between work and employment [4]. If we simply regard sustainable employability as a personal characteristic rather than resulting from the interaction between the working environment, the labour market, and individuals, we ignore important differences between the similar concepts of sustainable employability and sustainable workability. Furthermore, although the definition of Fleuren et al. applies to some unemployed individuals, it ignores older or retired individuals. Because the authors measure sustainable employability according to the nine aspects listed above, they ignore the role of the individual’s opportunity to enter the labour market (an environmental characteristic). The employability of an older or retired individual may be overestimated. Although these groups (for example, medical staff and teachers) may maintain a high working ability due to the accumulation of knowledge and experience [15], organisations might discriminate against older workers by showing them less appreciation and investing fewer resources in them. This would significantly reduce their chances of entering the labour market [16,17]. Therefore, even if older or retired individuals have the ability to work but cannot find employment, their actual employability is relatively low. However, as this variable is not considered in the definition of Fleuren et al., the sustainable employability of these groups according to this definition may be overestimated, suggesting that the definition cannot be widely applied to all groups. As the starting point of the original definition of van der Klink et al., work value has been mentioned many times in subsequent papers as a conceptual dimension of sustainable employability [18]. However, Fleuren et al. did not discuss or mention it in the process of establishing their definition. This has led to doubts about its accuracy. Therefore, there are some indicators missing in the mode of measurement proposed by Fleuren et al. Lastly, although there is a basis for the selection of each of the nine measurement indices proposed by Fleuren et al., the logical connection between the indices is not strong, and it is hard to classify them.

A key feature of recent changes in the labour market is that information and communication technologies (ICT) play an increasingly important role in several aspects of employment [19]. These technologies have affected the nature and employment situation of many industries and occupations, and their use will affect the location and time of work [20]. They underpinned the development in job search, recruitment, and selection practices [21]. Therefore, if employees want to make employability sustainable, they have to consider the impact of the digital age. Whether employees have digital exclusion determines whether they can adapt to this digital age and continue to work. However, the previous definitions of sustainable employability rarely emphasized the background of the times, indicating the importance of ICT.

Therefore, to solve the aforementioned problems, this paper redefines sustainable employability and its measurement dimensions by taking into account environmental factors, broadening its application to retired and elderly employees, incorporating work value, and simplifying the measurement method of Fleuren et al. for greater ease in future research. This will facilitate the study of intervention measures for sustainable employability and ultimately help resolve the future labour shortage caused by population aging.

## 3. Establishing a New Sustainable Employability Concept

### 3.1. Sustainable and Intrinsic Work Value

The adjective sustainable is used to describe something that is ‘able to continue at the same level for a period of time’ [22]. Finkbeiner et al. also point out that sustainability is at the original level. Specifically, it means that the resources are maintained after use, and the total amount has not decreased or even increased, which implies positive development and added value for the environment and stakeholders [23]. Similarly, PubMed defines sustainable development as ‘a process of change in which the exploitation of resources, the direction of investments, the orientation of technological development, and institutional change are all in harmony and enhance both current and future potential to meet human needs and aspirations’ [24].

As far as the sustainability of employability is concerned, we define employability to be sustainable if workers perceive that their work or work environment is valuable [1]. Jonathan Holslag’s The Strength of Paradise showed that work has become trivial and unattractive to many people in modern society. He advocated paying more attention to the values that are vital to human survival, arguing that values such as meaning and recognition can be satisfied in the workplace and can thus motivate people to continue working [25]. According to the theory of self-depletion, a person must consume resources when performing volitional activities (e.g., process control, active choice, initiation behaviour, and overcoming reactions), but such resources are often limited. The more abundant the resources available to perform volitional activities, the easier it is to succeed. For employees to successfully achieve sustainable employment and maintain a high level of sustainable employability, employees’ intrinsic motivation is needed as a resource for consumption [26]. According to self-determination theory, the satisfaction of basic psychological needs is very important for individual intrinsic motivation [27]. A valuable job can satisfy employees’ psychological needs of autonomy, competence, and psychological relatedness, thus promoting their intrinsic motivation [28]. In other words, according to the self-determination theory, the intrinsic work value that employees think can bring them intrinsic motivation. Combined with the theory of self-depletion, intrinsic work value, as a motivating factor, can be used as a resource to help employees maintain and even increase the original employability. Intrinsic work value connects self-depletion theory and the self-determination theory and injects ‘sustainable’ into employability. Studies have shown that when employees consider work as meaningful and can provide them with recognition, they are more likely to maintain their employability to increase their job security [29]. At the same time, empirical research has shown that intrinsic work value has a strong positive correlation with the three indicators of sustainable employment for employees of all ages (i.e., workers’ employability, work engagement, and affective commitment) [28].

In summary, by combining self-depletion theory with self-determination theory, we explain the impact of work value on employment sustainability as proposed by Jonathan Holslag and add intrinsic work value into the definition of sustainable employability as the dimension that makes employability sustainable.

### 3.2. Employability and Dynamic Chain

Employability was originally defined by Hillage and Pollard as a person’s ability to obtain and maintain employment and productivity [30]. In fact, from ‘maintaining and obtaining’, we can infer the concept of sustainability. In subsequent development studies, employability has been defined as individuals’ job opportunities in the internal or external labour market [31]. Based on this definition, scholars have examined what constitutes this kind of ‘opportunity’. Some have evaluated the realisation of job opportunities from the perspective of mobility (job transitions), others have focused on how personal advantages such as knowledge, skills, and attitudes influence job opportunities (movement capital), while others have explored the personal evaluation of job opportunities (perceived employability). Due to these different approaches, employability has become a vague and catch-all concept. To resolve this confusing situation, Forrier connected different concepts of employability into a ‘dynamic chain’ of three dimensions, namely job transitions, movement capital, and perceived employability [32]. Job transitions expand a person’s movement capital [33], movement capital improves a person’s perceived employability [34], while perceived employability encourages employees to achieve further job transitions [35].

### 3.3. Sustainable Employability and the swAge-Model

The swAge-model is considered to be a tool in the task of understanding how to make working life more sustainable and healthier for all ages, which can be the basis of sustainable employability [36,37]. The swAge-model describe three influence levels of importance for work life participation and to a sustainable extended working life: the individual level, micro level; the organizational and enterprise level, meso level; and the society level, macro level. Based on swAge-model, we have formed a more logical and informed definition of sustainable employability.

**Figure 1:**
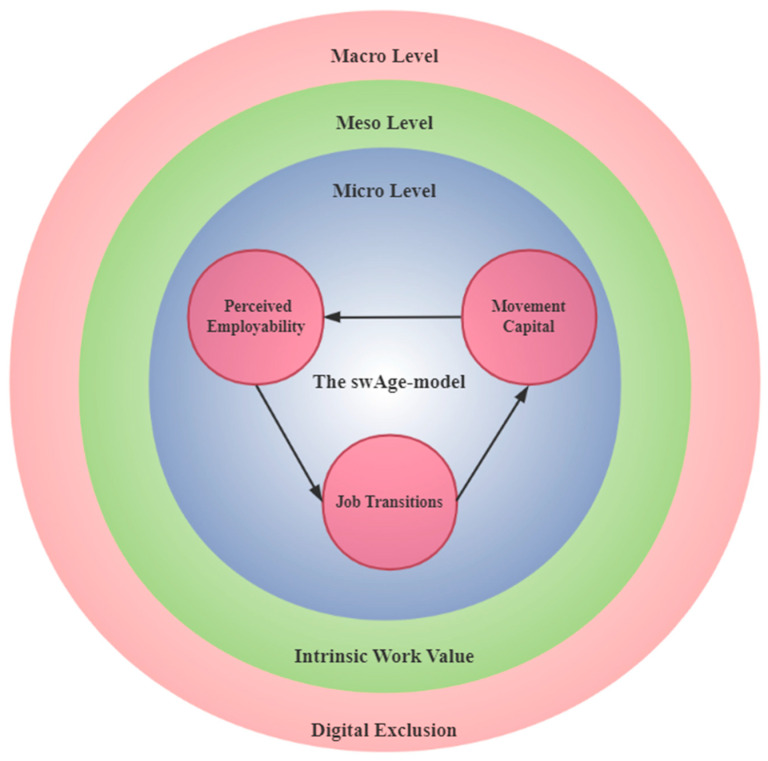
The dimensions of sustainable employability.

At the macro level, as mentioned earlier, the digital age has a huge impact on the labour market. ICT has a profound impact on the sustainability of employability, so digital exclusion should be used as one of the dimensions of sustainable employability. At the meso level, based on the original framework of van der Klink et al., we have selected intrinsic work value’ as one of the dimensions of sustainable employability, which makes employees more willing and able to continue employment and achieve sustainability in employability as well. At the micro level, combined with the dynamic chain, the three dimensions are included in the definition of sustainable employability, since they are related by mutual influence and promotion in a way that allows sustainability: through the interactions among the three dimensions, employability can remain unchanged or even increase over a period of time. The dimensions of this definition can be seen in Figure 1. Therefore, we define sustainable employability as follows:

‘In the digital age, sustainable employability refers to the ability of individuals who pursue work with high intrinsic value and avoid digital exclusion, remain in employment during their lifetimes. They expand personal movement capital through job transitions to improve their perception of employability, which encourages them to further achieve job transitions. The three components of job transitions, movement capital, and perceived employability are constantly promoted in a circular way, such that individuals can maintain or improve their original job opportunities in the labour market’.

First, this definition shows clear measurement dimensions including digital exclusion, intrinsic work value, job transitions, movement capital, and perceived employability, which facilitates the unification of subsequent measurement methods and the horizontal comparison of different studies later. Second, this definition considers that sustainable employability is a characteristic of the interaction between environment and individual, taking into account a person’s opportunities in the labour market, and meaning that this definition is better adapted to older or retired employees and prevents overestimation of their employability. Finally, this definition is based on the swAge-model, which takes into account the characteristics of the digital age, and combines the framework of van der Klink et al. and dynamic chain, which is simpler, more logical and more reliable than previous dimensions.

## 4. Components of Sustainable Employability

### 4.1. Digital Exclusion

Digital exclusion is broadly defined as being unable to access or use internet-enabled technology and Web-based services [38]. It can be divided into three aspects including access, motivation, and low confidence [19]. Access includes issues of physical access to hardware and software and affordability thereof. Motivation refers to encompassing lack of interest in or lack of perceived need to use ICT. Low confidence mainly means that employees lack confidence to adapt to the digital age and ICT [38].

### 4.2. Intrinsic Work Value

When the task is regarded as meaningful, challenging, and conducive to personal development, and when employees are recognised for their contributions, the work is regarded as having intrinsic value [28]. Recent research has focused on employees’ views on four aspects of work: meaning, recognition, challenge, and learning value. Work is considered meaningful when it provides a sense of accomplishment, purpose, and contribution [39]. Recognition means that a person’s contribution to their organisation is acknowledged and is usually regarded as one of the intangible rewards that motivate employees [40]. Challenge refers to when difficult task elements require employees to exert their potential by stimulating their curiosity, creativity, and enjoyment [41]. Learning value refers to the learning experience present in the working environment [42], which leads to the development of employees’ abilities [43].

### 4.3. Job Transitions

Job transitions represent an individual’s opportunities in the labour market and include any change in employment situation or substantial changes in job content [44]. These changes can be within the same organisation (internal work transfer), or across different organisations (external work transfer). Specific measurement indices can be divided according to whether internal work transfer or external work transfer is involved [32]. They can also be divided along the line of horizontal work transfer and vertical work transfer [45].

### 4.4. Movement Capital

Personal advantages increase employees’ opportunities in the labour market because they can help individuals effectively cope with labour market changes [46]. Movement capital accounts for these different personal advantages; that is, ‘personal skills, knowledge, abilities, and attitudes that affect career mobility’ [47]. It is usually divided into four dimensions: human capital, social capital, self-awareness, and adaptability. Human capital refers to an individual’s ability to meet specific professional performance expectations [48]. Social capital reflects the value of social networks in one’s career [49]. Self-awareness refers to the reflection on one’s past and present career and provides direction for future career opportunities [48]. Finally, adaptability refers to the necessary changes in behaviour, emotion, and thought to meet the requirements of the environment [50]. These four dimensions explain employability from the perspective of a person, which has received great attention in previous research [51].

### 4.5. Perceived Employability

Perceived employability explores employability from the perspective of personal views on existing employment opportunities. Advocates believe that it captures the interaction between personal and environmental factors, because people consider environmental factors such as labour market conditions in addition to personal factors when evaluating their employability [52]. The perception of employability can be determined in relation to current employers (perceived internal employability) or other employers (perceived external employability). These two dimensions are often put forward by other studies and used in empirical studies [53].

## 5. Discussion

This definition of sustainable employability solves leftover problems from the past. It confirms that sustainable employability is not a personal characteristic, but results from the interaction between individuals and the environment. In recent decades, access to Internet technologies and Web-based services has grown exponentially [19]. Whether employees can adapt to this digital age and avoid digital exclusion determines whether their future employability is sustainable [54]. Therefore, it is very important to emphasize the background of the times in the definition and add relevant dimensions. This further shows that governments can intervene in individuals’ sustainable employability through relevant policies, and that organisations should pay attention to the value of employees at work. An individual’s sustainable employability may be improved by increasing individual employment opportunities at an environmental level, rather than simply through individual efforts. This broadens the research scope of sustainable employability, develops more ways to increase employees’ working years, and solves the problem of future labour market shortages.

In addition, this concept of sustainable employability can apply to retired and older people. These groups are very important part of the medical staff. Some medical staff are relatively old, and their physical functions may have declined, but they are experienced and skilled, and they can still continue to engage in medical-related work [15]. It is important to reactivate the labour force comprising these groups so that we can solve the problem of labour shortages caused by the aging of the current population, especially in the medical sector [8]. To be more precise, our definition and measurement dimensions can evaluate the sustainable employability of these groups to help select relatively high-ability individuals for re-employment, increase their resources for entering the labour market, and ultimately solve the problem of talent shortage.

Furthermore, the new definition provides clear measurement dimensions, thus preventing confusion and incomparability between different studies. Moreover, the establishment of measurement dimensions is conducive to the development of quantitative research, which was called for in a recent qualitative study [55]. Subsequent intervention research on sustainable employability can provide a theoretical reference for enterprises on how to improve employees’ sustainable employability and increase their employment years. At the same time, our delineation of measurement dimensions is beneficial to research on sustainable employability in itself, and gives more practical significance to research in this field.

The sWage-model provides three dimensions: the individual level, micro level; the organizational and enterprise level, meso level; and the society level, macro level. We take this model as the basis for the definition and measurement dimensions of sustainable employability. Digital exclusion provides the background for definition at the macro level. Combining with the dynamic chain, we provide employability from the micro level to the definition. Combining self-determination theory and self-depletion theory, we explain the important role of intrinsic work value in the sustainability of employability from the meso level. This not only expands the content of the original theoretical framework, but also broadens their application scope. In other words, we combine different theories and models in our concept of sustainable employability and apply them in the field of management.

## 6. Limitations and Future Directions

Like all studies, this study has limitations. This article is preliminary work aiming to put forward the definition of sustainable employability and a few measurement dimensions, but cannot determine the specific measurement method. We suggest that descriptive, exploratory, structural, and other research methods can be used for further exploration. To be more precise, first, develop relevant scales based on the dimensions we have established, and verify the reliability and validity of the scales. Then carry out research on the mechanism of sustainable employability to explore its influencing factors and possible impacts. In the end, it will improve the sustainable employability of employees, expand the supply of the labour market, and solve the labour shortage. We have tried our best to review and evaluate and sustainable employability related researches, but there must be other researches that we have not noticed. Subsequent research can expand the scale of literature review and evaluation, and improve our existing definitions and dimensions.

## 7. Conclusions

We are in a period of population aging during which the labour shortage problem has gradually appeared. Although sustainable employability can prolong the working lives of employees and solve the problem of labour market shortages, the definition and measurement dimensions of this ability are unclear. The latest definition by Fleuren at al. improves on the generality of previous definitions to some extent, but there are still omissions in their conceptualisation. By defining sustainable employability as a personal characteristic, they ignore work value and environmental factors (e.g., labour market, digital age, etc.), which creates confusion with the concept of sustainable working ability and narrows the scope of research. Their neglect of environmental factors also means that retired or older employees are not well considered and measured in the original conceptual framework. Finally, the measurement method proposed by Fleuren et al. comprises nine indicators, but they do not form a logical framework; they are merely a collection of indicators, which is not convincing enough. Therefore, based on the swAge-model, this paper puts forward a new definition of sustainable employability that combines self-depletion theory and self-determination theory to explain the sustainability of employability. This definition makes the measurement dimensions clear and simplifies them as well. It improves the logical connection of the constituent components, helps researchers solve the problems present in the framework of Fleuren et al., facilitates later quantitative research, and provides information and a theoretical reference for future governments and enterprises on how to cope with population aging and labour market shortages.
